# Significant associations of PAI-1 genetic polymorphisms with osteonecrosis of the femoral head

**DOI:** 10.1186/1471-2474-12-160

**Published:** 2011-07-14

**Authors:** Hye- Ok Kim, Chang- Hoon Cho, Yoon- Je Cho, Seong- Ho Cho, Kyung- Sik Yoon, Kang- Il Kim

**Affiliations:** 1 Department of Biochemistry and Molecular Biology (BK21 project), Kyung Hee University School of Medicine, Seoul, 130-701, Korea; 2 MRC for Bioreaction to ROS and Biomedical Science Institute Kyung Hee University School of Medicine, Seoul, 130-701, Korea; 3 Department of Orthopaedic Surgery, Kyung Hee University Hospital at Gangdong, School of Medicine, Kyung Hee University, Seoul, 134-727, Korea; 4 Division of Allergy-Immunology, Department of Medicine, Feinberg School of Medicine, Northwestern University, Chicago, IL, USA

## Abstract

**Background:**

The pathogenesis of osteonecrosis of the femoral head (ONFH) has been implicated in hypofibrinolysis and blood supply interruption. Previous studies have demonstrated that decreased fibrinolytic activity due to elevated plasminogen activator inhibitor-1 (PAI-1) levels correlates with ONFH pathogenesis. The -675 4G/5G single nucleotide polymorphism (SNP rs1799889) in the PAI-1 gene promoter is associated with PAI-1 plasma level. We investigated whether rs1799889 and two other SNPs of the PAI-1 gene (rs2227631, -844 G/A in the promoter; rs11178, +10700 C/T in the 3'UTR) are associated with increased ONFH risk.

**Methods:**

Three SNPs in PAI-1 were genotyped in 206 ONFH patients and 251 control subjects, using direct sequencing and a TaqMan^® ^5' allelic discrimination assay. We performed association analysis for genotyped SNPs and haplotypes with ONFH.

**Results:**

The 4G allele of rs1799889, A allele of rs2227631, and C allele of rs11178 were significantly associated with increased ONFH risk (p = 0.03, p = 0.003, and p = 0.002, respectively). When we divided the population according to gender, an association between the three SNPs and increased risk of ONFH was found only in men. In another subgroup analysis based on the etiology of ONFH, rs2227631 (A allele) and rs11178 (C allele) in the idiopathic subgroup (p = 0.007 and p = 0.021) and rs1799889 (4G allele) and rs11178 (C allele) in the alcohol-induced subgroup (p = 0.042 and p = 0.015) were associated with increased risk of ONFH. In addition, a certain haplotype (A-4G-C) of PAI-1 was also significantly associated with ONFH (p < 0.001).

**Conclusion:**

Our findings demonstrated that three SNPs (rs1799889, rs2227631, and rs11178) of the PAI-1 gene were associated with ONFH risk. This study also suggests that PAI-1 SNPs may play an important role in ONFH.

## Background

Osteonecrosis of the femoral head (ONFH) is a devastating bone disease in which patients experience progressive collapse of the femoral head caused by a disturbance in the supply of blood and anomalies in the fibrinolytic system [1,2]. An increased tendency for intravascular coagulation is proposed as the pathogenetic mechanism responsible for interruption of the osseous blood supply and ONFH, and a significantly higher prevalence of coagulation abnormalities is reported in patients with ONFH [1,3]. Recent studies have suggested that genetic polymorphisms in factor V, prothrombin, methylenetetrahydrofolate reductase (MTHFR), and plasminogen activator inhibitor-1 (PAI-1) genes leading to intravascular coagulation disorders may be related to ONFH [4-6].

PAI-1 is a critical factor that regulates coagulation and fibrinolytic systems. Reduced plasma fibrinolytic activity, mainly attributable to increased levels of PAI-1, is associated with ONFH development [7,8]. Previously, PAI-1 levels were reported to be regulated by a common transcription-altering insertion/deletion single nucleotide polymorphism (SNP; rs1799889) of four or five guanine (4G/5G) nucleotides that is 675 bp upstream of the transcription start site. Homozygous or heterozygous carriage of the 4G allele is associated with higher PAI-1 levels [9]. In myocardiac infarction, subjects who are homozygous for the 4G allele (4G/4G genotype) have plasma PAI-1 concentrations that are approximately 25% higher than those of subjects who are homozygous for the 5G allele (5G/5G genotype) [10]. Glueck et al. first reported that the genotype frequency of 4G/4G was 41% in ONFH patients and 20% in healthy control subjects [8]. Moreover, Ferrari et al. reported a significant increase in the frequency of the 4G/4G genotype in renal transplant patients with ONFH compared to that of controls (60.3% vs. 20.6%) [7]. However, Asano et al. suggested that plasma PAI-1 levels are highest in ONFH patients with the 4G/4G genotype, but that the incidence of ONFH is not related to genotype [11]. To better understand the genetic influences of PAI-1 on ONFH, we selected SNPs in the promoter and 3'UTR that are known to be involved in the regulation of gene expression. To determine whether PAI-1 SNPs, including 4G/5G, are associated with susceptibility to ONFH, genotype and allele frequencies were analyzed. We also investigated whether pathological etiology (idiopathic, alcohol- or steroid-induced) is involved in the association of SNPs and ONFH.

## Methods

### Patients and controls

A total of 206 (159 men, 47 women; mean age 44.2 ± 11.6) patients with non-traumatic ONFH who had total hip arthroplasty at Kyunghee University Hospital (Seoul, Korea) were consecutively enrolled in this study. The diagnosis of symptomatic ONFH was based on anteroposterior and lateral pelvic radiography and magnetic resonance images. Patients were subgrouped according to etiological factors into idiopathic (98 cases), steroid-induced (72 cases), and alcohol-induced (36 cases) ONFH. Patients with a demonstrable history of direct trauma or with possible combined causes were excluded. Steroid-induced ONFH was defined by a history of taking prednisolone 1800 mg or the equivalent over 4 weeks with nephritic syndrome, and organ transplantation [12]; and alcohol-induced ONFH was diagnosed based on the consumption of more than 400 ml of alcohol per week [13]. Patient characteristics are summarized in Table 1.

**Table 1:** Characteristics of ONFH patients and controls

	Controls	Patients			
		
	(n = 251)	Total (n = 206)	Idiopathic (n = 98)	Alcohol-induced (n = 72)	Steroid-induced (n = 36)
Age (year)^a^	47.8 ± 12.6	44.2 ± 11.6	44.9 ± 12.3	45.7 ± 10.2	39.3 ± 10.9
Sex (M/F)	193/58	159/47	78/20	62/10	19/17
BMI (kg/m^2^)^a^	23.6 ± 3.5	24.2 ± 3.3	24.4 ± 3.6	24.1 ± 2.9	24.4 ± 3.3

^a^Mean ± SEM

Controls were recruited from subjects attending routine medical checkups at our institution who had no coagulation-related disorder or other chronic disease, such as diabetes or cardiovascular disease (CHD). Control subjects were matched with patients with regard to gender and age (193 men, 58 women; mean age 47.8 ± 12.6). This study was approved by the Institutional Review Board at our hospital, and informed consent was obtained from all patients.

### Polymerase chain reaction and genotyping

Genomic DNA was isolated from peripheral blood leukocytes using an AxyPrep Blood Genomic DNA Miniprep kit (Axygen Biosciences, Union, CA, USA). The ~1.0 kb promoter region of the PAI-1 gene was partially amplified using PCR and analyzed via direct sequencing. All PCR reactions were in a 20 μl volume containing 1.5 mmol/L MgCl_2_, 40 mmol/L KCl, 10 mmol/L Tris-HCl (pH 9.0), 250 μmol/L dNTP, 1 U Taq DNA polymerase (Bioneer, Daejeon, Korea) and 50 ng genomic DNA in distilled water. The forward primer 5' GTG CTT GAA TCA TCC CGA AAC 3' and reverse primer 5' TCT GGA CCA CCT CCA GGA AA 3' were used for amplification. The conditions for the PCR reaction were denaturation at 95°C for 10 min, followed by 35 cycles of denaturation at 95°C for 30 sec, annealing at 58°C for 30 sec, and extension at 72°C for 30 sec, followed by a final extension at 72°C for 10 min. PCR products purified with 95% ethyl alcohol were used as template DNA for cycle sequencing. The PCR for sequencing was performed using a Big Dye Terminator (ver 3.1) cycle sequencer and analyzed using an ABI Prism^® ^3730 Automated DNA sequencer (Applied Biosystems, Foster City, CA, USA).

The SNP of the PAI-1 3'UTR (rs11178) was analyzed with primers and probes for TaqMan^® ^SNP genotyping assays. Primer Express (Applied Biosystems, Foster City, CA, USA) was used to design both the PCR primers and the MGB TaqMan^® ^probes. One allelic probe was labeled with 6-carboxyl-fluorescent (FAM)™ dye and the other was labeled with fluorescent VIC^® ^dye. PCRs were carried out in TaqMan^® ^Universal Master mix without UNG (Uracil-N-Glycosylase; Applied Biosystems), containing PCR primer concentrations of 900 nM and TaqMan^® ^MGB-probe concentrations of 200 nM. Reactions occurred in a 96-well plate with a total reaction volumes of 10 μl using 20 ng of genomic DNA. Plates were placed in a thermal cycler (7300 SDS 1.2.2, Applied Biosystems, Foster City, CA, USA) and heated at 50°C for 2 min and 95°C for 10 min, followed by 40 cycles at 92°C for 15 sec and 60°C for 1 min, with post-reading at 60°C for 1 min. Fluorescence data files were analyzed using automated allele-calling software (SDS 2.1, Applied Biosystems, Foster City, CA, USA). Genotyping quality control was performed in 10% of samples via duplicate checking (rate of concordance in duplicates > 99%).

### Statistical analysis

We tested significant deviations in genotype frequency from Hardy-Weinberg equilibrium (HWE) at each polymorphic variant using the χ^2 ^test. Odds ratios (ORs) and 95% confidence intervals (CIs) were used to estimate the relative risks of ONFH patients associated with the presence of different PAI-1 genotypes. We employed a widely used measure of linkage disequilibrium (LD) between all pairs of biallelic loci, D', and r^2^. Haplotype structures and their frequencies were estimated from genotyped data using Haploview http://www.broad.mit.edu/mpg/haploview based on the expectation maximization (EM) algorithm. The χ^2 ^test was used to compare the frequencies of discrete variables between ONFH patients and controls. All statistical analyses were performed using SPSS for Windows version 16.0 and p-values less than 0.05 were regarded as significant. All statistical tests were two-sided.

## Results

To identify if PAI-1 SNPs were involved in susceptibility to ONFH, three SNPs in the promoter and 3'UTR were selected based on a minor allele frequency (MAF) > 0.05 and Hardy-Weinberg equilibrium (HWE) > 0.05 using a public database http://www.ncbi.nlm.nih.gov/SNP/. Two SNPs (rs2227631, -844 G/A; rs1799889, -675 4G/5G) in the promoter region and one (rs11178, +10700 C/T) in the 3'UTR of the PAI-1 gene were genotyped in 206 ONFH and 251 control subjects (Table 2).

**Table 2:** Frequencies of PAI-1 polymorphisms (n = 457)

Position^a^	Location		Genotype			MAF^c^	Heterozygosity
g.-844G/A	promoter	GG	GA	AA	N	0.387	0.425
(rs2227631)		183	194	80	457		

g.-6754G/5G	promoter	4G4G	4G5G	5G5G	N	0.410	0.492
(rs1799889)		157	225	75	457		

g.10700	3'-UTR^b^	CC	CT	TT	N	0.500	0.457
(rs11178)		124	209	124	457		

^a^rs No. was shown if present in the dbSNP database; ^b^3'-untranslated region; ^c^frequencies of minor alleles

The p value of each polymorphism was determined for comparison between ONFH patients and controls. The rs2227631, rs1799889, and rs11178 genotypes and/or allele frequencies of the PAI-1 gene were significantly associated with ONFH risk (p = 0.002 - 0.03). These results suggest that the minor alleles of rs2227631 (A), rs1799889 (4G), and rs11178 (C) in the PAI-1 gene contribute to an increase in ONFH risk (Table 3).

**Table 3:** Genotypes and allelic frequencies of PAI-1 gene polymorphisms between ONFH patients (n = 206) and controls (n = 251)

Position	Genotype/Allele	Controls, n (%)	Patients, n (%)	OR (95% CI)	p
g.-844G/A	GG	116(46.2)	67(32.5)	1*	**0.011**
(rs2227631)	GA	97(38.6)	97(47.1)	1.73(1.15-2.61)	
	AA	38(15.1)	42(20.4)	1.91(1.12-3.26)	
	G	329(65.5)	231(56.1)	1*	**0.003**
	A	173(34.5)	181(43.9)	1.49(1.14-1.95)	

g.-6754G/5G	5G5G	46(18.3)	29(14.1)	1*	0.073
(rs1799889)	4G5G	130(51.8)	95(46.1)	1.16(0.68-1.98)	
	4G4G	75(29.9)	82(39.8)	1.73(0.99-3.04)	
	5G	222(44.2)	153(37.1)	1*	**0.030**
	4G	280(55.8)	259(62.9)	1.34(1.03-1.75)	

g.10700C/T	TT	79(31.5)	45(21.8)	1*	**0.013**
(rs11178)	CT	116(46.2)	93(45.1)	1.41(0.89-2.22)	
	CC	56(22.3)	68(33.0)	2.13(1.28-3.55)	
	T	274(54.6)	183(44.4)	1*	**0.002**
	C	228(45.4)	229(55.6)	1.50(1.16-1.95)	

*reference category (odds ratio, 1.0)

Further analysis based on pathological etiology (idiopathic, alcohol- or steroid-induced) showed that the allele frequencies of PAI-1 (rs2227631 and rs11178) between idiopathic ONFH patients and controls were significantly associated (p = 0.007 and 0.021). In addition, the allele frequencies of rs1799889 and rs11178 were significantly associated with the alcohol-induced ONFH subgroup (p = 0.042 and 0.015, respectively). These results suggest that the PAI-1 SNPs were important risk factors in idiopathic (rs2227631, rs11178) and alcohol-induced (rs1799889, rs11178) ONFH (Table 4). In addition, the association of PAI-1 polymorphisms was further analyzed in the ONFH subgroup stratified according to gender. Interestingly, the genotype frequencies of rs2227631, rs1799889, and rs11178 were significantly associated with ONFH in men but not in women (Table 5). These results suggest that the PAI-1 SNPs might be important genetic factors in ONFH susceptibility in men.

**Table 4:** Genotypes and allelic frequencies of PAI-1 gene polymorphisms between ONFH subtype patients (n = 206) and controls (n = 251)

			Patients			p		
				
Position	Genotype	Controls	Idi^a^	Alc^b^	Ste^c^	vs Idi	Alc	Ste
g.-844G/A	G	329(65.5)	107(54.6)	83(57.6)	41(56.9)	**0.007**	0.082	0.154
(rs2227631)	A	173(34.5)	89(45.4)	61(42.4)	31(43.1)			
	GG+GA	213(84.9)	75(76.5)	61(84.7)	28(77.8)	0.066	0.977	0.279
	AA	38(15.1)	23(23.5)	11(15.3)	8(22.2)			

g.-6754G/5G	5G	222(44.2)	76(38.8)	50(34.7)	27(37.5)	0.191	**0.042**	0.282
(rs1799889)	4G	280(55.8)	120(61.2)	94(65.3)	45(62.5)			
	4G5G+5G5G	176(70.1)	61(62.2)	41(56.9)	22(61.1)	0.157	**0.036**	0.274
	4G4G	75(29.9)	37(37.8)	31(43.1)	14(38.9)			

g.10700C/T	T	274(54.6)	88(44.9)	62(43.1)	33(45.8)	**0.021**	**0.015**	0.164
(rs11178)	C	228(45.4)	108(55.1)	82(56.9)	39(54.2)			
	TT+CT	195(77.7)	66(67.3)	47(65.3)	25(69.4)	**0.046**	**0.032**	0.274
	CC	56(22.3)	32(32.7)	25(34.7)	11(30.6)			

^a^idiopatic ONFH; ^b^alcohol-induced ONFH; ^c^steroid-induced ONFH

**Table 5:** Genotype frequencies of PAI-1 gene polymorphisms between ONFH patients and controls in men and women

		Men			Women		
			
Position	Genotype	Controls	Patients	p	Controls	Patients	p
g.-844G/A	GG	92(47.7)	53(33.3)	**0.025**	24(41.4)	14(29.8)	0.386
(rs2227631)	GA	71(36.8)	74(46.5)		26(44.8)	23(48.9)	
	AA	30(15.5)	32(20.1)		8(13.8)	10(21.3)	

g.-6754G/5G	5G5G	37(19.2)	21(13.2)	**0.030**	9(15.5)	8(17.0)	0.976
(rs1799889)	4G5G	102(52.8)	73(45.9)		28(48.8)	22(46.8)	
	4G4G	54(28.0)	65(40.9)		21(36.2)	17(36.2)	

g.10700C/T	TT	62(32.1)	33(20.8)	**0.003**	17(29.3)	12(25.5)	0.718
(rs11178)	CT	91(47.2)	69(43.4)		25(43.1)	24(51.1)	
	CC	40(20.7)	57(35.8)		16(27.6)	11(23.4)	

We analyzed linkage LDs among the PAI-1 SNPs and did not observe complete LD for the PAI-1 gene. LD between SNPs was estimated by calculating the D' values (Table 6). We also calculated the haplotype frequencies of rs2227631, rs1799889, and rs11178 in ONFH patients and controls. The distribution of haplotype A-4G-C was significantly different between the ONFH patients and controls (p < 0.001) (Table 7). These results suggest that these SNPs should be further analyzed in future studies regarding PAI-1 functions and ONFH.

**Table 6:** LD coefficients (|D' |) and r^2 ^among polymorphisms in the PAI-1 gene

			|D'|	
	SNPs	rs2227631	rs1799889	rs11178
r^2^	rs2227631	-	0.932	0.786
	rs1799889	0.382	-	0.786
	rs11178	0.391	0.43	-

**Table 7:** Haplotype frequencies of PAI-1 gene polymorphisms between ONFH patients (n = 206) and controls (n = 251)

	Haplotype			Frequency			
				
	rs2227631	rs1799889	rs11178	Controls	Patients	OR (95% CI)	p
Hap1^a^	G	5G	T	0.391	0.310	1*	-
Hap2	A	4G	C	0.305	0.398	1.63(1.31-2.02)	**<0.001**
Hap3	G	4G	C	0.113	0.103	1.14(0.83-1.55)	0.425
Hap4	G	4G	T	0.115	0.092	0.98(0.71-1.34)	0.875

^a^Haplotype G-5G-T (Hap1) was chosen to be the baseline haplotype; *reference category (odds ratio, 1.0)

## Discussion

We determined the contributions of PAI-1 gene SNPs to ONFH. We identified for the first time a significant association between SNPs of the PAI-1 gene (rs2227631; -844 G/A, rs1799889; -675 4G/5G, rs11178; +10700 C/T) and ONFH in Koreans. The A, 4G, and C alleles considerably increased disease risk (Table 3).

PAI-1 is a fast-acting inhibitor of fibrinolysis, and increased plasma levels are associated with increased incidence of thrombophilia [14] and osteonecrosis [8,11,15,16]. High levels of PAI-1, induced by -675 4G/5G SNP in the PAI-1 promoter, lead to suppression of fibrinolysis through inhibition of plasminogen activator and promotion of thrombosis. The resulting increase in intraosseous venous pressure which restricts flow to the femoral head may culminate in osteonecrosis [8,16]. The PAI-1 gene is reported to be polymorphic, especially in rs1799889 (-675 4G/5G) of the promoter region. The possible association between rs1799889 and osteonecrosis risk has been studied, leading to controversial results. Glueck et al. and Ferrari et al. reported that the 4G allele is a major predisposing factor in ONFH patients [7,8], but the findings of Asano et al. were contradictory to that conclusion [11]. Glueck et al. showed that, in Americans, twice as many patients as healthy control subjects (41% vs. 20%) were homozygous for 4G/4G, and 19% of patients and 36% of the control subjects had the 5G/5G genotype (p = 0.001) [7]. Furthermore, Ferrari P. et al. observed that in 228 glucocorticoid-treated renal transplant patient in Switzerland, the prevalence of ONFH according to genotype was 1.8% for 5G/5G, 7.7% for 4G/5G, and 30.3% for 4G/4G (p < 0.001 vs. 4G/5G and 5G/5G); the prevalence of ONFH according to genotype in subjects with persistent hyperparathyroidism was 4.2% for 5G/5G, 15.2% for 4G/5G, and 55.5% for 4G/4G (p < 0.003 vs. 4G/5G and p < 0.001 vs. 5G/5G) [8]. Asano et al. studied 31 Japanese patients with postrenal transplant ONFH and found four patients with 5G/5G, 11 with 4G/5G, and 16 with 4G/4G. However, analysis revealed no significant differences in the incidence of ONFH among these patients (p = 0.49) [11].

In addition to rs1799889, another SNP in the promoter region of PAI-gene, rs2227631 (-844 G/A), is potentially implicated in PAI-1 gene regulation. To date, a significant association has been reported between this SNP and coronary heart disease (CHD) in nonsmokers, with patients having a higher frequency of rs2227631 A allele. In addition to rs2227631 and rs1799889, rs11178 (+10700 C/T) also significantly associates with increased CHD risk in nonsmokers. The correlation between rs11178 and CHD in nonsmokers might be attributable to strong linkage LD of this SNP with rs2227631 and rs1799889 [17]. For ONFH, the results of our study confirmed an association between rs1799889 and disease risk in a Korean population. Moreover, for the first time, we showed that rs2227631 and rs11178 are essential SNPs involved in the regulation of PAI-1 gene expression in ONFH (Table 3).

Several transcription factor binding sites for PAI-1 are found in the 5' and 3' UTR regions, and the transcriptional regulation of the gene is extremely complex [18]. Several studies have shown that SNPs within the 5'UTR lead to differences in PAI-1 expression between individuals, and this could influence the etiology of a variety of pathological conditions with which PAI-1 is associated such as cancer, rheumatoid arthritis and stroke [19-21]. We classified ONFH into two or three major subgroups based on etiology and gender: idiopathic, alcohol-induced, steroid-induced groups, and men and women. We found that the risk effects of rs2227631 and rs11178 in the idiopathic subgroup, rs1799889 and rs11178 in the alcohol-induced subgroup, and all three SNPs in men were significantly associated with ONFH. However, no association was seen in the steroid-induced group or in women. Glucocorticoid has been reported to increase PAI-1 activity and is a potential risk factor for ONFH development [7,22]. However, we had limited data on SNPs of PAI-1 in steroid-induced ONFH. The incidence of ONFH is relatively low and gender-biased. We examined 206 cases, of which 159 (77%) were men. Moreover, epidemiologic analysis showed that the incidence of steroid-induced ONFH in overall ONFH was low (17.5%). In Korean studies, the proportion of steroid-induced ONFH was previously shown to be small (range, 12.6% to 15.4%) [23,24]. We found that, in Koreans, steroid-induced ONFH is more rare than idiopathic or alcohol-induced ONFH. Thus, we found no significant association between PAI-1 SNPs and steroid-induced ONFH because of the small sample size. In addition, differences according to subgroup could not be clearly distinguished. Therefore, the association analysis strategy of subgrouping according to gender or etiology has limitations. However, the results of the steroid-induced group showed a similar tendency as those of the other groups, demonstrating that patients more often have risk alleles (A, 4G, and C) than do control subjects, although this finding was not significant (Table 4 and 5). Thus, we suggest that PAI-1 SNPs are involved in ONFH risk in Korean patients. To firmly establish the relationship between the PAI-1 SNPs and steroid-induced ONFH, further study with larger sample sizes is required.

The pathophysiology of ONFH is not well known, although a number of polymorphisms in candidate genes (HIF-1, VEGF, eNOS, IL23R, SREBP-2, ANXA6) were recently identified in an attempt to determine the genetic factors involved in ONFH pathogenesis in a Korean population [24-29]. Some studies have suggested that genetic polymorphisms leading to thrombosis (factor V, prothrombin, MTHFR) may be related to ONFH [4,6,16,30,31]. Intravascular coagulopathy including thrombotic and fibrinolytic abnormalities may play an etiologic role in the disease, and studies have investigated the association between ONFH and genes involved in the coagulation and fibrinolytic system [7,11,32].

## Conclusion

We found that SNPs of the PAI-1 gene, which is involved in coagulation, were significantly correlated with ONFH. These data suggest that PAI-1, along with already reported candidate genes, may be useful genetic markers to identify high-risk individuals in Korea. The results of this association study suggest that PAI-1 gene polymorphisms may be important genetic factors in ONFH susceptibility in a Korean population. To further substantiate this hypothesis, functional studies of PAI-1 regulation are required. The polymorphisms analyzed in this study may contribute to further studies on the function of PAI-1 and the development of ONFH.

## Abbreviations

ONFH: (osteonecrosis of the femoral head); PAI-1: (plasminogen activator inhibitor-1); SNP: (single nucleotide polymorphism).

## Competing interests

The authors declare that they have no competing interests.

## Authors' contributions

KIK and KSY conceived the study, and supervised the study design. HOK, CHC and YJC were involved in the design of the genotyping assays, sample preparation, and statistical analysis. KIK, HOK and SHC contributed to interpretation of the data. KIK drafted the manuscript together with HOK, CHC, YJC, SHC and KSY. All authors read and approved the final manuscript.

## Pre-publication history

The pre-publication history for this paper can be accessed here:

http://www.biomedcentral.com/1471-2474/12/160/prepub
